# Optical nanostructures in 2D for wide-diameter and broadband beam collimation

**DOI:** 10.1038/srep18767

**Published:** 2016-01-06

**Authors:** James Clark, José V. Anguita, Ying Chen, S. Ravi P. Silva

**Affiliations:** 1 Advanced Technology Institute, University of Surrey, Guildford, Surrey, GU2 7XH, United Kingdom

## Abstract

Eliminating curved refracting lensing components used in conventional projection, imaging and sensing optical assemblies, is critical to enable compactness and miniaturisation of optical devices. A suitable means is replacing refracting lenses with two-dimensional optical media in flat-slab form, to achieve an equivalent optical result. One approach, which has been the focus of intense research, uses a Veselago lens which features a negative-index metamaterial. However, practical implementations rely on resonance techniques, thus broadband operation at optical frequencies imposes significant technical challenges that have been difficult to overcome. Here, we demonstrate a highly-collimated, broadband, wide-diameter beam from a compact source in flat-slab form, based on light collimation using nanomaterials ordered in patterns and embedded into flexible polymers. These provide a highly anisotropic absorption coefficient due to patterns created by vertical carbon nanotube structures grown on glass, and the anisotropic electrical conductivity of the nanotubes. We show this nanostructure strongly absorbs unwanted off-axis light rays, whilst transmitting the desired on-axis rays, to achieve the required optical effect over broadband, from visible to short-infrared, thus circumventing some technical limitations of negative-index metamaterials. We further show a low substrate-temperature system for nanotube growth, allowing direct implementation into heat-sensitive large-area devices.

Replacing the bulky refractive components used in current optical assemblies with thin two-dimensional (2D) media in flat-slab form is a key enabler for the integration of complex optical systems into single compact units. This ability for integration introduces significant commercial advantages such as eliminating the requirement for current lensing components, reducing the size and weight of the component, in addition to opening up the possibility for implementing flexible optical components. Moreover, the elimination of refractive components offers the potential to eliminate spectral restrictions introduced by the spectral transparency of the optical materials used, and also eliminates wavelength dispersion of the index of refraction. These practical restrictions are accentuated for the case of applications requiring wide-beam collimation assemblies that use a single light source, since these require a wide-diameter lens (shown as the red component in Fig. 1(a)), such as in projection, microscopy, imaging, and photolithography projections.

One approach is to replace the refractive convex lens with a 2D lensing material in the shape of a flat-slab of material. Veselago introduced this concept decades ago using a hypothetical “left-handed” medium which features an isotropic index of refraction of −1, known as a Veselago Lens^1^,^2^. Such a medium achieves its negative index by featuring negative values for its electric and magnetic polarization responses, occurring simultaneously within at least, a particular range of “useful” wavelengths. However, left-handed materials do not occur naturally, and instead artificial “metamaterials” that feature inductive and capacitive components in the unit cells (for example using split-ring resonators) need to be produced. These structures are designed to resonate with the excitation wave to generate the negative magnetic and electric responses, around the excitation wavefrequency^2^,^3^.

However, metamaterials require unit cell dimensions throughout the bulk that are smaller than the wavelength they operate at. For this reason, isotropic behaviour in three-dimensions (3D) has only yet been achieved for metamaterials of centimetre-scale dimensions. These metamaterials operate at quite specific narrow bandwidth frequencies such as in the case of microwave frequencies^3^,^4^,^5^,^6^. Operating at higher frequencies (towards the visible) requires scaling down the component dimensions into the nanoscale, which demands significant technical fabrication challenges that have been difficult to overcome. For this reason, scaling down has only been possible for metamaterials with reduced dimensions (2D or 1D). This scaling has enabled operation in the THz regime^7^,^8^ and more recently in the infrared^8^. Similar resonance-based approaches using periodic arrays of parallel pairs of gold nanorods^9^, pairs of dielectric voids in metal^10^ and nano-fishnet^11^ have resulted in operation around 1.5 μm and near-infrared region^9^,^10^,^11^,^12^. More recently, parallel approaches using stacked plasmonic waveguides that are strongly coupled have enabled negative behaviour in the UV^13^,^14^. However, for all these cases, the resonance-based method results in the negative index behaviour being observed only over a narrow range of wavelengths. More recently a phase-compensated medium using diffractive and refractive components combined into a single metasurface has been demonstrated, in order to broaden the operation bandwidth of the 2D medium^15^. This compensation achieves still narrow bandwidth lensing behaviour at three discrete wavelengths between 1300–1800 nm^15^. However, broadband operation of metamaterials throughout the visible range remains difficult, due to the resonant nature of their operation. In addition, the transmitted beam features strong losses during transmission, originating from resonating currents in metals at high frequencies, and strong decay of surface plasmons during propagation. These pose significant barriers to practical implementations that operate at visible frequencies.

Here we present an alternative approach to negative-index lensing, where we produce a large-area flat-slab optical material that is right-handed (positive index of refraction) but is also able to produce a collimated beam of light. The material is suitable for wide-beam optical assemblies by virtue of its ability to selectively absorb unwanted (off-axis) rays, resulting in effective beam collimation, Fig. 1(b). A requirement for this implementation is the transition from the use of a single source of light, Fig. 1(a), to using a 2D array of light sources (or detectors, for the case of a detector array). Fig. 1(b) shows that this non-lensing implementation eliminates the need for the wide-diameter convex-shaped lens (shown as the red component in Fig. 1(a)) thereby alleviating the aforementioned restrictions associated with the use of wide-diameter refractive lenses. Our alternative method is based on engineering a nanostructure to enable an absorption coefficient that is highly anisotropic, a field of study which is currently relatively unexplored in terms of practical applications. This anisotropy enables the material to strongly absorb light that is off-axis to the nanostructures within the material (off-axis from the normal to the slab), whilst enabling strong optical transmission of the rays that are on-axis to the nanostructure (on-axis to the normal to the slab). We present the design, production and implementation of our 2D slab for broadband beam collimation, operating at all wavelengths from the visible to the short-infrared. We also highlight its simplicity in production, modelling and mechanism of operation, compared to using flat-lensing by left-handed metamaterials. Furthermore, a low-temperature route for the synthesis of the nanostructure is presented, which potentially enables growth of the nanostructure directly onto a 2D array of light sources. This in turn would allow integration of the collimator and light source array into one single compact device, which will open new avenues in compactness, for example, wafer-thin projectors. Such integration enables significant weight, size and cost reductions by virtue of eliminating the need for the wide-diameter lens, (red component in Fig. 1a), as well as eliminating the spectral restrictions introduced by the material for this lens.

In this paper we experimentally produce and demonstrate a flat slab of this material. We show that a flat-slab of this material that is only 150 μm thick, is able to appear mainly transparent to the naked eye (enabling the transmission of visible light) when the viewing on-axis to the nanostructure (“on – mode”). Simultaneously, the material appears completely opaque (strongly absorbing) when the nanostructure is off-axis (“off-mode”) to viewing axis, even by only a few degrees. We show that in the “off-mode”, the nanostructure is able to fully absorb even intense light such as that from a laser beam. Our experimental and computational analysis show the origin of this strong contrast in transmission between the “on” and “off” orientation stems from the strongly anisotropic electrical conductivity of the material, which is provided by a forest of multiwalled carbon nanotubes (MWCNTs)^16^,^17^,^18^,^19^. The small diameter of the MWCNTs (~20 nm) is significantly smaller than the wavelength of visible light, which is unable to resolve individual tubes and as a result, the beam experiences an effective anisotropic metamaterial medium^20^.

In addition to the broadband operation characteristics of our material, we further show a unique method for MWCNT growth that enables direct implementation into existing commercial components. This is in contrast to conventional growth techniques that rely on high substrate temperatures to grow the MWCNTs (such as furnaces, etc). Our growth technique is based around top-down optical heating using a photo-thermal chemical vapour deposition (PT-CVD) system that features back-cooling of the substrate^21^,^22^,^23^. This enables growth on heat-sensitive substrates, enabling processing compatibility with semiconductor materials. Substrates include glass, or semiconductor wafers containing complementary metal-oxide-semiconductor (CMOS) circuits, such as those with pixelated source/detector arrays, where the pixels require optical isolation by the MWCNT vertical nanostructures^24^,^25^. This optical isolation is an increasing necessity particularly for cameras operating at long wavelengths in the infrared, where the pixel dimension is comparable to the wavelength^25^. We further show the nanostructure can be generated over relatively large-area glass substrates (4 cm^2^ here), transferred, and encapsulated within a thin layer of transparent flexible polymer, polydimethylsiloxane (PDMS). This can then be peeled-off from the glass-substrate to produce a free-standing, flexible form of the collimator that is low-cost, moreover it can even be cut-and-pasted into curved geometries to generate alternative optical effects.

We highlight that the operation principle of our nanostructure is different from that of left-handed metamaterials. Yet the ability for integration introduced by our flat-slab material provides significant commonality with the benefits from using a hypothetical Veselago flat lens at visible wavelengths, from a practical perspective, without the restrictions of a narrow range of resonant frequencies^8^,^9^,^10^,^11^,^12^,^13^,^14^. We believe this technology will find a wide range of applications in optical and holography systems, particularly those where compactness & line-of-sight is essential.

## Results

### Material Fabrication

Low substrate-temperature growth of MWCNT forest structures was performed using a PT-CVD system described in detail elsewhere^21^,^22^,^23^. The process used is the same as that used for production of Vantablack™ films. In brief, infrared lamps situated above the substrate table provide the thermal energy required for CNT nucleation and growth, Fig. 2(a). The substrate is placed on a silicon carrier wafer which is mounted on a water-cooled substrate table. This arrangement provides cooling to the back of the substrate, which enables growth on heat-sensitive substrates. The glass substrate is mainly transparent to the optical radiation, which is a key enabler for CNT growth on glass substrates using this approach, Fig. 2. The catalyst regions are on the top of the glass substrate, which absorb the incident radiation, causing localised heating necessary for the growth of high-quality CNTs over large areas. The thermal model in Fig. 2(b) illustrates the MWCNT structures at the top of the glass substrate exhibit significantly higher temperature than the bulk of the glass substrate due to this thermal design.

The patterned MWCNT forests were grown for a period of 10 minutes, to a forest height of 150 μm. The average growth rate of the forest, 15 μm min^−1^, is significantly higher than previously reported for growth of CNT on glass substrates, as a result of the localised heating to the catalyst regions, directly at the growth front of the CNTs, coupled with the low thermal conductivity of glass^26^,^27^,^28^. The ability to grow highly vertical and aligned walls of MWCNTs to produce tall structures is shown in Fig. 3(a–d). This pattern was reproducible over large areas, with reproducible forest height throughout the sample. We report this method to produce highly aligned CNT walls is greatly suited to generate features with a high aspect ratio, in this case, forest height of 150 μm and a line width of 2 μm, Fig. 3(c).

The quality of the forest was ascertained by non-destructive Raman spectroscopy, using the 514 nm line of an argon-ion laser, Fig. 3(f). The spectrum reveals the presence of multiwalled CNTs, deduced from the absence of the radial breathing modes (150 to 300 cm^−1^) which are associated with single-walled CNTs. Also no splitting in the G-band (1582 cm^−1^) is observed^29^. The high crystalline quality of the tubes is ascertained from the prominent second-order peaks (~2700 cm^−1^), of similar intensity to the G-band, which show long-range order, indicative of a highly crystalline sp^2^ carbon structure^30^,^31^,^32^.

**Figure 1:**
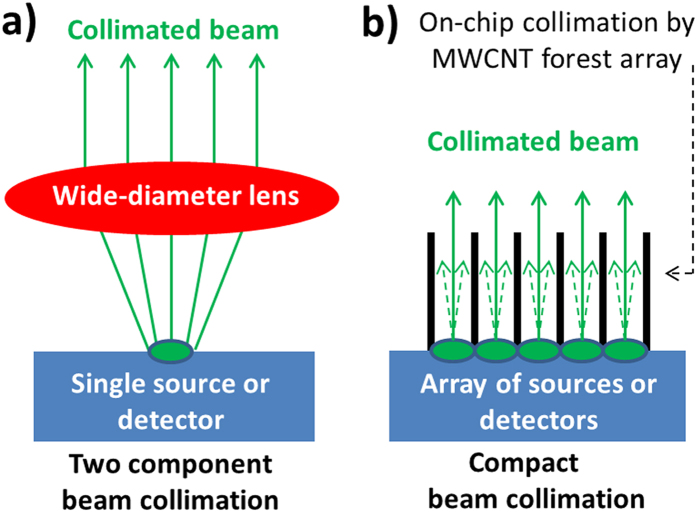
Ray diagrams comparing two optical assemblies used to deliver a wide-beam and collimated source of light for projection purposes. (**a**) Conventional (two-component) projector featuring a light source (green) and a wide-diameter convex lens (in red), and (**b**) Compact (wafer-thin) source of collimated light, featuring no lensing requirements, using an array of light sources and the nanostructured collimator in the same optical component. The MWCNT forest structures could feasibly be grown directly on the array of sources (or detectors, for the case of sensing) using a low-temperature PTCVD growth process, which enables the compact design. Unwanted off-axis beams are absorbed by the nanostructure, depicted with the dashed arrows.

**Figure 2:**
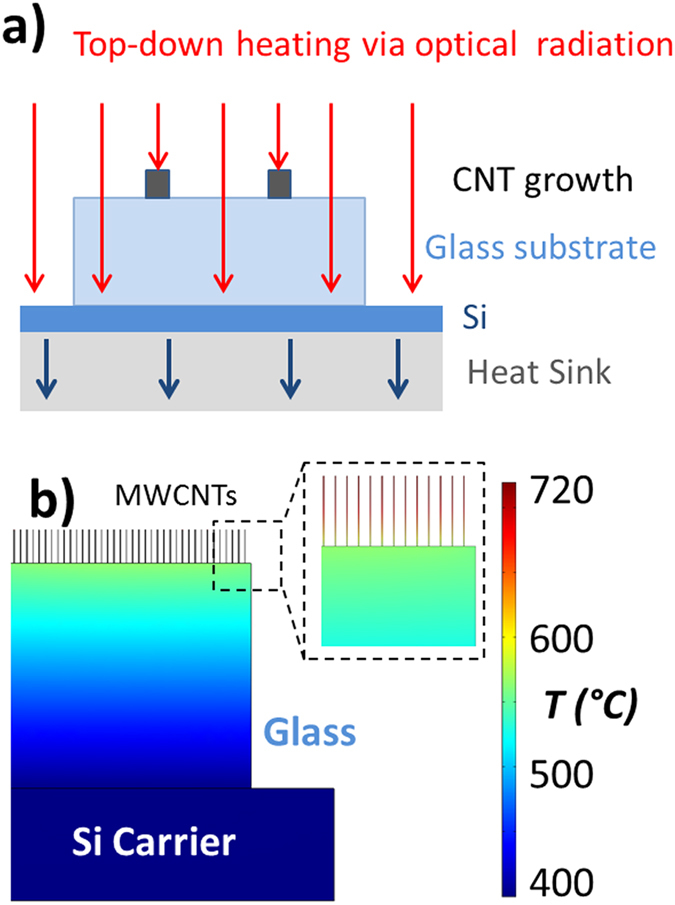
Description of top-down optical heating arrangement used to grow the MWCNT vertical structures on glass. (**a**) Ray-diagram schematic showing optical radiation (red arrows) absorbed by the nanotube forests structures (black) on top of the glass substrate, whilst the radiation transmits through the glass and is absorbed by the silicon/heatsink substrate table. (**b**) Thermal model of temperature profile of sample (steady-state) during the growth process. The figure shows the glass remains at a significantly lower temperature than the CNT structures, which is a key enabler of this method for CNT growth using heat-sensitive substrates such as glass.

**Figure 3:**
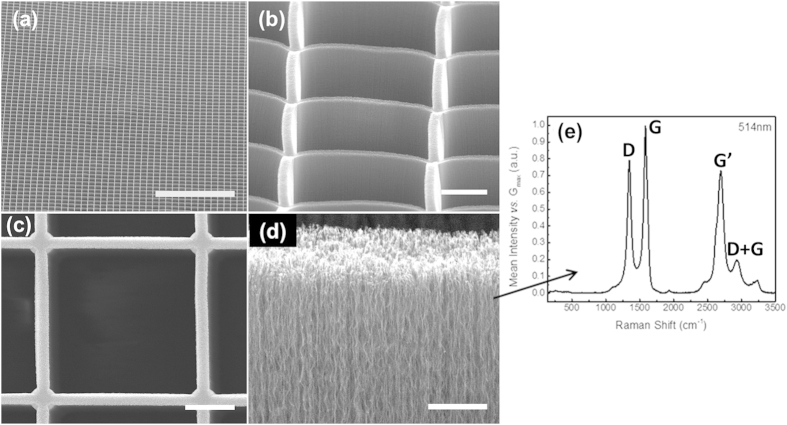
Structure of MWCNT vertical walls used for the optical collimator grown directly on glass, showing. (**a**) Square pattern over a large area, scale-bar is 400 μm, 55° tilt angle, (**b**) high aspect ratio and verticality of wall side-profile, scale-bar 15 μm, 55° tilt angle (**c**) Top-down view showing verticality of wall forest (150 μm tall), scale-bar 10 μm, 0° tilt angle, and (**d**) Detailed imaging of nanotubes within the wall, scale-bar is 2 μm, 80° tilt angle. (**e**) Raman spectra of MWCNT walls grown on glass, using a laser wavelength of 514 nm. The narrow peaks and prominent G and G′ peaks (of similar intensity) indicate a high degree of crystallinity within the sp^2^ carbon structure.

The finite-element computer model, Fig. 2(b), shows the maximum temperature reached by the substrate (at steady-state) appears close to the top surface, and is circa 500 °C. This lower temperature, compared to that used in conventional CVD growth, enables growth of MWCNT structures directly on heat-sensitive substrates such as glass. In addition, our group has reported elsewhere^33^ the use of optical mirrors and heat-shielding layers to reduce further the substrate temperature to only 350 °C. This enables MWCNT growth on heat-sensitive semiconductors featuring opto-electronic CMOS components, such as detector/emitter pixelated arrays. The lower growth temperature reduces the risk of uncontrolled dopant diffusion at elevated temperatures (450 °C+). This top-down heating arrangement enables the PT-CVD system to be directly implemented into MWCNT optical designs such as our collimator, and thus into existing devices to enable lens-free compact designs, Fig. 1(b).

### Optical Collimation Experiments

**Figure 4:**
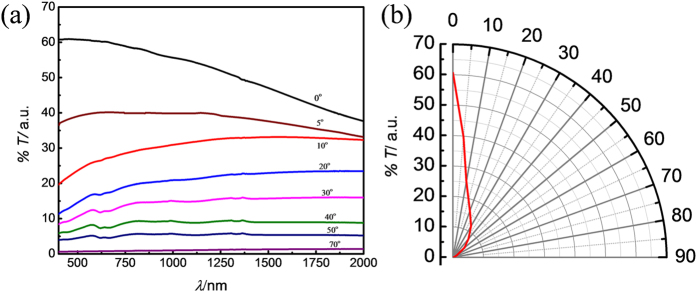
Optical angular performance of nanostructured MWCNT collimator on glass. (**a**) Transmission spectrum (from the visible to the short-infrared) at various angles of incidence to the beam in the spectrometer, from normal incidence (0° black curve), exhibiting maximum transmittance, to 70°, indicating near-zero transmittance (high absorption). (**b**) Angular dependency of the transmission at 550 nm (green visible light) highlighting the strong dependency of the transmission as a function of angle of incidence. The transmission is reduced sharply upon small deviations to the angle of incidence from the normal. This highlights the high performance of the nanostructure for beam-collimation purposes.

The transmission of the sample was measured from the visible to the short-infrared, as a function of the angle of incidence to the beam, from 0° (normal incidence) to 70°, Fig. 4(a), as a suitable means to measure the spectral transparency of the samples as a function of angle of incidence. The glass substrate is mostly transparent within this spectral range. A pristine glass substrate was used to obtain the background for baseline subtraction. The transmission spectrum obtained at normal incidence shows an average transmission value ~ 50%, with small variations in the transmission across this wide spectral range, of around ± 10%. It was observed that the transmission is strongly reduced across all wavelengths by increasing the angle of incidence to the beam, reaching almost 0% transmission at an angle of 70°. The strong dependence of the transmission on the angle of incidence is highlighted in the polar diagram in Fig. 4(b) for a wavelength of 550 nm. This strong dependency on the beam orientation is highly desirable for optical applications that require strong transmission of the desired rays (on-axis with the nanostructure) whilst strongly absorbing other unwanted rays that are off-axis to the nanostructure, such as for beam collimation. This behaviour is observed across a broadband spectral range, including the visible. We also observe that using an incidence angle of 5° results in a near wavelength-independent transmission across this wide spectral range, Fig. 4(a), elucidating the possibility for neutral-density designs.

**Figure 5:**
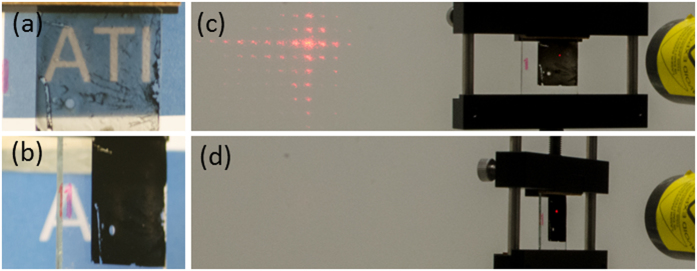
Photographic illustration of the optical contrast performance of the MWCNT collimator on glass. (**a**) Visual transparency of MWCNT collimator when viewing at normal incidence, 0° enabling the viewing of the “ATI” logo behind the collimator, and (**b**) blocking of the transmission when the collimator is tilted to an angle of 45° to the line of sight. (**c**) Transmission of an intense HeNe laser (632.8 nm) beam at normal incidence to the collimator, and (**d**) complete blocking of the intense beam when the collimator is at 45°.

This strong optical anisotropy can be observed from the samples upon visual inspection, Fig. 5. In the figure, the sample appears transparent in transmission mode when viewing an object (“ATI” text in the figure) at normal incidence in the “on” – orientation, Fig. 5(a). This transparency is substantially reduced upon tilting the samples by only a few degrees. The samples lose their transparency and become totally opaque (absorbing) upon further increasing the angle of tilt to 45° or greater (“off - orientation”, Fig. 5(b)). This strong on-off contrast behaviour was further demonstrated using intense radiation from a 300 mW HeNe laser beam operating at 632.8 nm. The transparency of the sample to the laser beam in the “on” orientation is shown in Fig. 5(c). However, in the “off” orientation (45°, Fig. 5(d)), the sample is able to absorb all the light from the intense laser beam where the laser spot is visible on the MWCNT sample. The ability to completely block such an intense beam using only 150 μm tall MWCNT walls further highlights the strongly anisotropic optical absorption of the MWCNTs within the nanostructure as well as their utility as a good heat sink for this application.

### Optical Collimation Modelling

**Figure 6:**
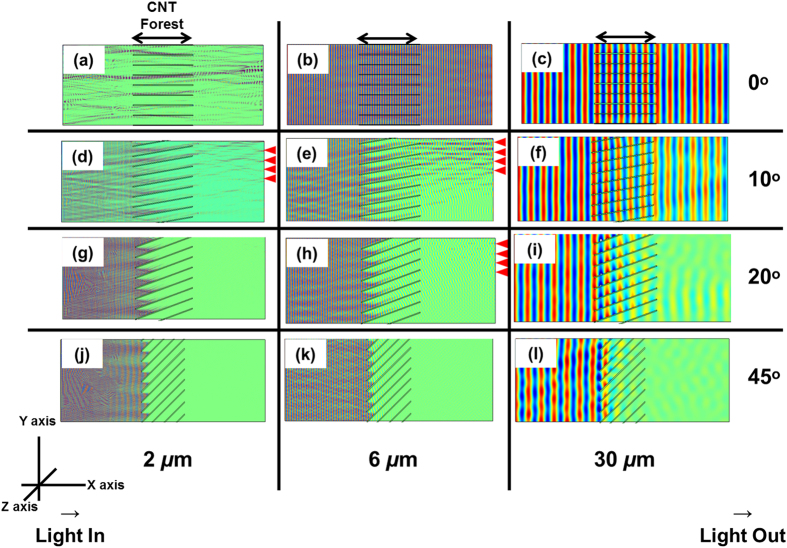
Computational Modelling of the propagation of optical radiation though the structure as a function of tilt angle (relative to the propagation direction of the incidence wave) for three wavelengths. (**a**–**c**) Normal incidence (0°) for the three wavelengths, indicating no appreciable attenuation of the wave. For this case, the collimator exhibits mainly transmission behaviour. (**d**–**f**) 10° of tilt to the direction of propagation. Substantial attenuation of the wave is observed, although a significant amount of the light is transmitted, particularly at the longer wavelengths. (**g**–**i**) 20° of tilt, indicating a significant wave attenuation, although some transmission is observed, and (**j**–**l**) 45° of tilt, showing the wave becomes fully attenuated (particularly at the shorter wavelengths) after travelling only ~10% of the length of the collimator. The arrows on the right of some of the figures indicate regions of constructive interference from the deflected and un-deflected beams, showing similar periodicity as the patterns.

Insight into the absorption mechanism as a function of tilt angle was obtained by modelling the transmission of radiation along a cross-section of the structure, for four different angles of tilt (0°, 10°, 20° and 45°) with respect to the incident wave, Fig. 6. The figure also shows this effect for three different wavelengths. For all cases, wave propagation is along the positive x-axis, the electric field is along the positive y-axis, and magnetic field along the positive z-axis in the figure. The figures display the intensity of the electric field of the wave, which is excited from the left side of the figures (along the y-axis), using an amplitude of 1 V m^−1^. The horizontal structures in the centre of each image represent a cross-section of the MWCNT wall structure, which is perpendicular to the electric field. For the finite-element model, these walls were approximated to be composed of a homogeneous “effective-medium” material, with properties that are linearly extrapolated from the properties of air and MWCNTs, and their volume ratio. For this, a CNT density of 10^9^ cm^−2^ was used, using a tube diameter of 20 nm, and bulk conductivity of 10^4^ S cm^−1^ along the CNTs axis (parallel to the 0002 plane), and 10 S cm^−1^ across the CNTs (perpendicular to the 0002 planes)^23^. The anisotropic electrical conductivity in this effective medium is provided by the anisotropic nature of the conductivity of MWCNTs.

From the model, it is observed that the attenuation of the wave is minimal when the tilt angle is zero degrees (Fig. 6). This is observed for all wavelengths modelled, and occurs as a result of the low electrical conductivity of the MWCNT forest in the transverse direction. This result is in qualitative agreement with the experimentally observed absorption at zero-tilt, Fig. 4, which results in the case with lowest absorption. The circa 60% transmission from the experimental measurement likely arises from deviations in tube verticality from 90° which infer additional absorption mechanisms that are not included in the model, in addition to imperfections in processing i.e. photolithography. Fig. 6 shows that using even a shallow angle of tilt (10° or 20°) results in a pronounced increase in the attenuation of the wave (Fig. 6 (d–j)). This occurs as a result of strong coupling between the electric field of the wave and the y-component of the electrical conductivity of the forests, which gives rise to the strong absorption. Increasing the tilt angle to 45° results in strong beam attenuation, as a result of the increasingly strong coupling between the electric field and the y-component of the conductivity of the nanostructure. For this case, the beam is completely attenuated even after travelling only a short distance (~10%) along the nanostructure. This is the observed result in the spectroscopic and laser transmission experiments, which show complete absorption of the intense laser beam at this, and higher angles of tilt, Fig. 5(d). The model suggests also that the attenuation is strongest at the shorter wavelengths. This is also in qualitative agreement with the experimental observation, Fig. 4(a), which shows generally higher absorption closer towards the shorter wavelengths of the spectrum.

Additionally, the model further suggests that for the shallow angles, and the shorter wavelengths, a proportion of the beam is deflected into the tilt direction. This deflected beam interferes with the transmitted (un-deflected) beam, and forms an interference pattern in the near-field. The regions of constructive interference are observed at the right-hand side of the models, and are indicated by the arrows in the figure. The periodicity of the arrows is similar to that of the pattern, which re-enforces the occurrence of a deflected beam due to diffraction events. The beam that is diffracted along the opposite direction to the tilt angle becomes strongly absorbed by the nanostructure^34^.

**Figure 7:**
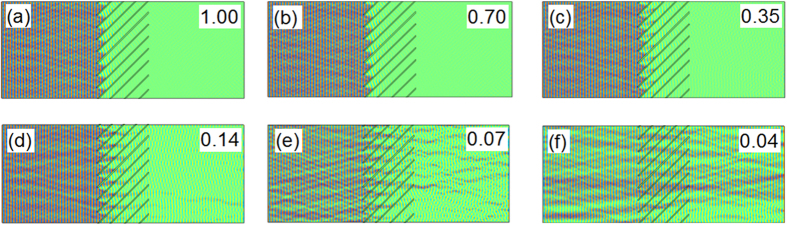
Simulation of the propagation of 6 μm wavelength radiation though the medium titled at an angle of 45° to the propagation direction, as a function of electrical conductivity of the medium (top-right for each case, ×10^4^ S cm^−1^). (**a**–**c**) The wave is fully absorbed by the medium at this angle of tilt. (**d**) Some transmission is observed when the conductivity is reduced by approx. 10× the original value. (**e**) Significant wave transmission occurs when the conductivity is reduced by ~15× the original value. (**f**) The structure becomes almost entirely transparent when the conductivity is reduced by ~25× from the original value. The model suggests that the ability to block the incident wave at an angle of tilt depends strongly on the electrical quality of the MWCNTs that are used to make the collimator device.

The nature of the wave coupling to the nanostructure can be ascertained from Fig. 7, which shows the absorption of the incident wave as a function of the electrical conductivity of the MWCNTs for a constant incidence angle of 45°. The figure shows strong coupling of the electric field relative to the structure when the structure exhibits high degrees of electrical conductivity, causing strong absorption, Fig. 7(a–c). Reducing the conductivity, Fig. 7(d–e) significantly reduces the ability of the nanostructure to absorb the incident (off-axis) wave, to the point of almost transparency of the structure, Fig. 7(f). This reinforces an absorption mechanism based on field coupling to the axis of the MWCNTs.

**Figure 8:**
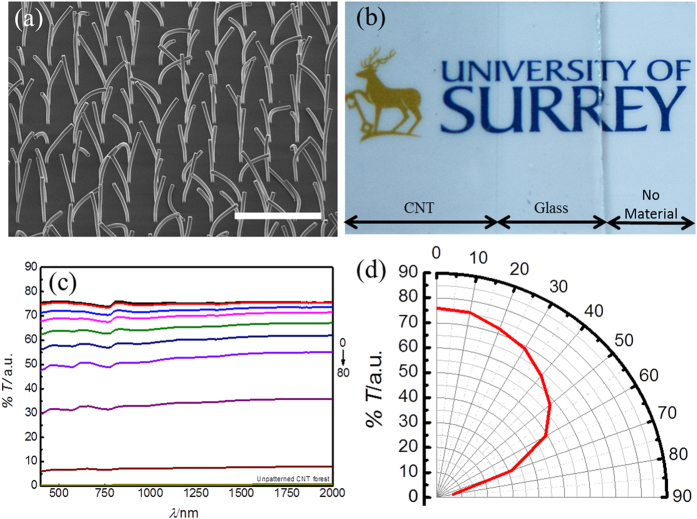
Nanopattern for collimator with a wider range of angles of acceptance. (**a**) Tall MWCNT pillars grown on glass, scale: 100 μm, tilt angle 55°. The pillars show some bending due to the narrow area of the base compared to the height of the tube bundle. (**b**) Photograph of the wider-angle collimator obtained at normal incidence (left of the image) comparing the bare glass substrate (centre) and the bare background image (right). The figure visibly shows significant transparency at normal incidence for this collimator design. (**c**) Transmission spectrum as a function of the angle of tilt relative to the incidence beam of the spectrometer, and (**d**) dependency of the transmission on the angle of incidence at 550 nm. Note the significantly higher transmission values than those in the pattern in Fig. 4, highlighting a wider range of transmission angles.

We have investigated in a similar manner a different nanostructure, composed of vertical rods of MWCNT bundles (Fig. 8). This design also exhibits anisotropic behaviour. However, for this design, the angular dependency of the transmission shows significantly higher transmission at wider angles compared to that from the structure in Fig. 4. This alternative performance may be suited to a different application. In this case, the transparency at normal incidence is ~75%, and is also wavelength independent, Fig. 8(c). Similarly to the design depicted in Fig. 4, the transparency is reduced with increasing tilt angle. However, for this design, the reduction of the transmitted intensity with increasing angle is lower than that for the previous design, and full beam absorption is only achieved at very high angles of tilt (~80°). This result indicates that the degree of optical collimation (and therefore angular dependence of the extinction coefficient) can be tailored to the particular application by simply modifying the nanostructure design.

**Figure 9:**
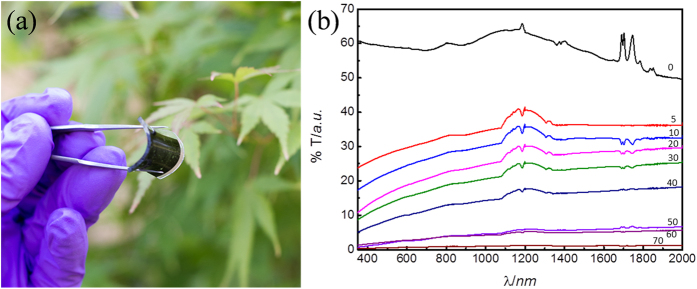
Transfer of collimator nanopattern into a flexible flat sheet of PDMS. (**a**) Illustration of nanopattern transferred and embedded into a flexible sheet of PDMS, highlighting its flexibility. (**b**) Transmission spectrum of nanopattern embedded into PDMS (flat sheet) as a function of angle of incidence. The peaks observed around 1200 nm and 1750 nm are characteristic absorptions of PDMS^35^, and due to differences between the PDMS used for baseline and measurement.

As well as growing the nanostructure on glass, we report this flat nanostructure can be encapsulated in an optically transparent polymer such as Polydimethylsiloxane (PDMS), to produce a flexible and low-cost version of the collimator. This is performed by covering the nanostructure on the glass substrate in the transparent PDMS fluid. Curing of the PDMS fluid results in a flexible polymer flat-slab layer that contains the MWCNT nanostructure. The polymer is then peeled away from the glass substrate, carrying the MWCNT structure, Fig. 9(a). The resulting thin flexible membrane retains the optical collimating characteristics, Fig. 9(b), and it can be directly applied to optical components. This procedure enables low-cost production and scalable physical dimensions (diameter), which accentuates the practical advantages compared to conventional convex lens manufacturing.

## Discussion

The experimental results and the model highlight the strong capability of the MWCNT nanostructures to modulate the intensity of the transmitted beam as a function of angle of incidence. This occurs by virtue of modulating the degree of coupling between the electric field of the incident beam to the anisotropic electrical conductivity of the nanostructure. Such anisotropic conductivity is provided by the specific design of the nanostructure, and also by the inherent one-dimensional conductivity along the axis, provided by the MWCNTs. This combination enables the strong “on-off” contrast ratio of the intensity of the transmitted and blocked beams, as a function of angle of incidence. This gives rise to a strong angular dependence of the extinction coefficient. This result is remarkable, given that the strong effects are observed using only a thin, 150 μm thin flat-slab of material. This contrast is a significant improvement over previous reports^19^ and originates as a result of the pattern used in this work. Although our 2D slab operates in a different way to a Veselago lens, our results show that there is commonality in their implementation into practical applications.

We have demonstrated the use of nanoscale positive index materials to reproduce some of the optical characteristics normally observed from negative index materials, albeit operating by a different mechanism. In particular, we show our flat-slab material has the ability to collimate a beam of light. Our low-cost approach is compatible with low substrate temperature growth of MWCNT structures over large areas, which is directly applicable, for example, to pixelated projection or CCD imaging applications, to provide pixel optical isolation. We have also shown that it is possible to tailor the nanostructure design to suit the optical requirements for a particular application. Finally, we have demonstrated the possibility to transfer the nanopattern into flexible transparent substrates, which opens new possibilities for novel optical designs.

## Methods

### Photolithography on Glass

Borosilicate microscope slide glass (Thermo-Fisher Scientific) was cut using a diamond scribe to 25 mm × 25 mm. The substrate was cleaned using sonication for 10 minutes in sequential diluted decon 90 (1:40 in DI water), iso-propanol, methanol and DI water. Substrates were O_2_ plasma ashed (Emitech K1050X) for 5 minutes at 100 W in 5 sccm O_2_. Substrates were patterned using photolithography, first HMDS primer was spun on at 3500 rpm for 10 s, followed by AZ-5214 photoresist at 4000 rpm for 30 seconds. Substrates were baked on a hot plate at 95 °C for 60 seconds and left to cool, this was followed by UV exposure (Karl Suss MA1006) with the appropriate mask for 6 seconds at 7 mW cm^−2^. Finally, substrates were developed in AZ 400K (1:4 with DI water) for 35 seconds, then washed thoroughly with DI water and dried using a N_2_ air gun. The photolithography mask pattern had 2 μm lines with a 30 μm pitch. DC-sputtering (JLS MPS) of thin metal films was used to deposit 7 nm Al followed by 3 nm Fe onto the substrates, using 25 sccm Ar at 5 mTorr. Lift-off was performed using NMP-1165 overnight.

### Carbon Nanotube Growth

CNTs were then grown on the substrates using PT-CVD (Surrey NanoSystems, model 1000N), samples were annealed at 47.5% power (3.8 kW) in 100 sccm H_2_ for 5 minutes at 2 Torr. This was followed by CNT growth at 47.5% power output in 164 sccm H_2_ and 36 sccm C_2_H_2_ for 10 minutes at 2 Torr.

### Sample Characterisation

Samples were characterised using scanning electron microscopy (FEI Quanta ESEM), UV-Vis-NIR spectroscopy (Varian Cary 5000 UV-Vis-NIR spectrophotometer) and Raman spectroscopy (Renishaw MicroRaman). A clean glass slide from the same pack was used for the baseline measurement in the UV-Vis-NIR spectroscopy.

### PDMS Replication

The substrate was replicated into PDMS, this was achieved by mixing PDMS with its crosslinker (1:10 ratio) and curing in a vacuum desiccator for 48 hours. The PDMS was removed by peeling from substrate using tweezers producing a clean replication.

## Additional Information

**Data Availability:** Details of the data and how to request access are available from the University of Surrey publications repository http://epubs.surrey.ac.uk/809519.

**How to cite this article**: Clark, J. *et al.* Optical nanostructures in 2D for wide-diameter and broadband beam collimation. *Sci. Rep.* **6**, 18767; doi: 10.1038/srep18767 (2016).
